# Death of Dr James Crawford Gregory

**Published:** 1833-02

**Authors:** 


### DEATH OF DR JAMES CRAWFORD GREGORY.

It is with sincere regret that we record the decease of Dr. James Crawford Gregory, which took place at Edinburgh, on the 28th ult. He was our contemporary, and son of the illustrious author of the "Conspectus Medicinæ Theoreticæ." Descended from a long line of distinguished professors, his father, grandfather, and great-grandfather, having filled the chairs of Mathematics, Institutes, and Practice of Medicine, in the Scottish Universities.

He inherited the abilities and talents, if we may use the phrase, of his justlycelebrated ancestors. He was a most assiduous and zealous student; and on him devolved the responsibility of supporting and perpetuating the renowned name of Gregory. After obtaining the doctor's degree in the University of Edinburgh, he repaired to France, and there we can attest he was most indefatigable in pursuit of science. We happened to be in Paris at the time, and felt sincere pleasure in observing his unceasing industry, as we, in common with all the disciples of his father, indulged in the gratifying anticipation that he would one day maintain the name of his revered ancestors. At this period the late Dr. William Cullen, nephew to the Scottish Hippocrates, was in Paris, and he, too, evinced the most satisfactory proofs that he was determined to follow in the wake of his renowned relation. These gentlemen published, in 1829, an edition of Dr. Cullen's First Lines of the Practice of Physic, and rendered the work accordant with the views of that period.

The scion of Cullen was cut off in the prime of life, and the representative of the illustrious philosophers of the Gregory family has shared a similar fate. Both would have added lustre to our Alma Mater had they been spared.

Dr. Gregory was one of the finest figures we ever saw. His features were regular, indeed truly handsome, and displayed an intelligence and a benevolence rarely observable. He was indefatigable as physician to the Royal Infirmary; he was the favorite pupil of the great Laennec; and he contributed, to the Edinburgh Medical and Surgical Journal, some valuable papers on diseases of the chest, and on diseases of the kidneys. His complaint was malignant typhus, with which he was said to be infected in performance of his duties at the Infirmary. So great was the malignity of the disease, that, though he was in the prime of life (his age being thirty-two) and had the assistance of Dr. Abercrombie and his cousin, Professor Alison, he sunk on the twelfth day of his illness.

Thus has Scotland to mourn, during 1832, among her illustrious dead, other victims of the king of terrors; Walter Scott, Leslie, Cullen, and Gregory have been gathered to their forefathers.

We are grieved to state, that Drs. Abercrombie and Alison, two of the ablest physicians of Edinburgh, are, according to the newspaper reports of the capital of Scotland, despaired of, (Monday, January 7th.) It is said, that there is a most malignant typhus now prevailing in Edinburgh.—*Med. and Surg. Journal.*

---

